# Congenital Inferior Vena Cava Atresia Unmasked by Pyrexia and Extensive Bilateral Deep Vein Thrombosis in a Young Adult With MTHFR Mutation

**DOI:** 10.7759/cureus.90081

**Published:** 2025-08-14

**Authors:** Daya Mani Jacob, Divyashri Ramanathan Nagarajan, Diana George, Rawan Mohamed, Niyas Khalid Ottu Para

**Affiliations:** 1 Internal Medicine, Burjeel Medical City, Abu Dhabi, ARE; 2 Radiology, Burjeel Medical City, Abu Dhabi, ARE

**Keywords:** deep vein thrmobosis, inferior vena cava (ivc) thrombosis, ivc atresia, pyrexia of unknown origin, pyrexia of unknown origin (puo)

## Abstract

A previously healthy, 22-year-old male presented with a one-month history of high-grade intermittent fever, low back pain, and unintentional weight loss. Initial workup revealed markedly elevated C-reactive protein and D-dimer levels with extensive bilateral deep vein thrombosis (DVT). Imaging studies identified atresia of the intrahepatic inferior vena cava (IVC) with prominent collateral venous circulation and associated thrombosis. Genetic testing revealed a methylenetetrahydrofolate reductase (MTHFR) mutation, contributing to a prothrombotic state. Despite empirical antibiotic therapy, the patient’s fever and weight loss persisted, which prompted further evaluation. CT pulmonary angiography revealed necrotic mediastinal lymphadenopathy concerning for lymphoma or granulomatous disease. This case highlights the diagnostic complexity of pyrexia of unknown origin (PUO) in young adults and underscores the importance of considering rare congenital vascular anomalies such as IVC atresia in patients presenting with bilateral DVT, especially when accompanied by systemic symptoms and other radiological features.

## Introduction

Pyrexia of unknown origin (PUO) remains one of the most challenging diagnostic dilemmas in clinical medicine, particularly among young adults. Defined as a persistent fever exceeding 38.3°C for more than three weeks without an identified source despite initial evaluation, PUO demands a broad differential diagnosis encompassing infectious, malignant, autoimmune, and miscellaneous causes [1]. While common etiologies such as tuberculosis or lymphoma frequently dominate consideration in endemic regions, rarer conditions such as congenital vascular anomalies must not be overlooked, especially when initial investigations are inconclusive.

Inferior vena cava (IVC) anomalies, such as atresia or agenesis, are uncommon congenital conditions that can remain asymptomatic until a triggering event reveals their clinical significance. Among young patients presenting with idiopathic or bilateral deep vein thrombosis (DVT), congenital IVC abnormalities are increasingly recognized as an important underlying cause. These anomalies can result in impaired venous return, the development of extensive collateral circulation, and a predisposition to thrombosis, often manifesting as atypical symptoms, such as low back pain or limb swelling [2].

Congenital absence or atresia of the IVC is estimated to occur in 0.0005% to 1% of the general population [3]. IVC defects are often discovered incidentally, either through imaging or during evaluations for thromboembolic events. Imaging studies have shown that approximately 5% to 6.7% of young adults aged 20 to 40 years who present with DVT have IVC anomalies, significantly higher than in the general population. Notably, 62.5% of these individuals had bilateral DVT, compared to just 8.6% in the broader group of acute DVT patients. This is likely due to bilateral venous stasis resulting from equal involvement of both lower limbs [4]. 

This report describes a previously healthy, 22-year-old male patient who presented with PUO, low back pain, bilateral DVT, and unintentional weight loss. The diagnostic journey revealed a rare combination of IVC atresia with extensive thrombosis and necrotic mediastinal lymphadenopathy, prompting consideration of both congenital and potentially malignant causes.

Additionally, the presence of a methylenetetrahydrofolate reductase (MTHFR) gene mutation adds further complexity. Congenital IVC atresia and MTHFR gene mutations are individually recognized risk factors for venous thromboembolism; however, their coexistence is exceedingly rare, with only a few cases reported in the literature, primarily in young adults presenting with extensive or bilateral DVT [5]. This case underscores the importance of considering vascular anomalies in young patients with unexplained thrombotic events and highlights the need for a multidisciplinary, imaging-guided approach in complex cases of PUO.

## Case presentation

**Figure 1:**
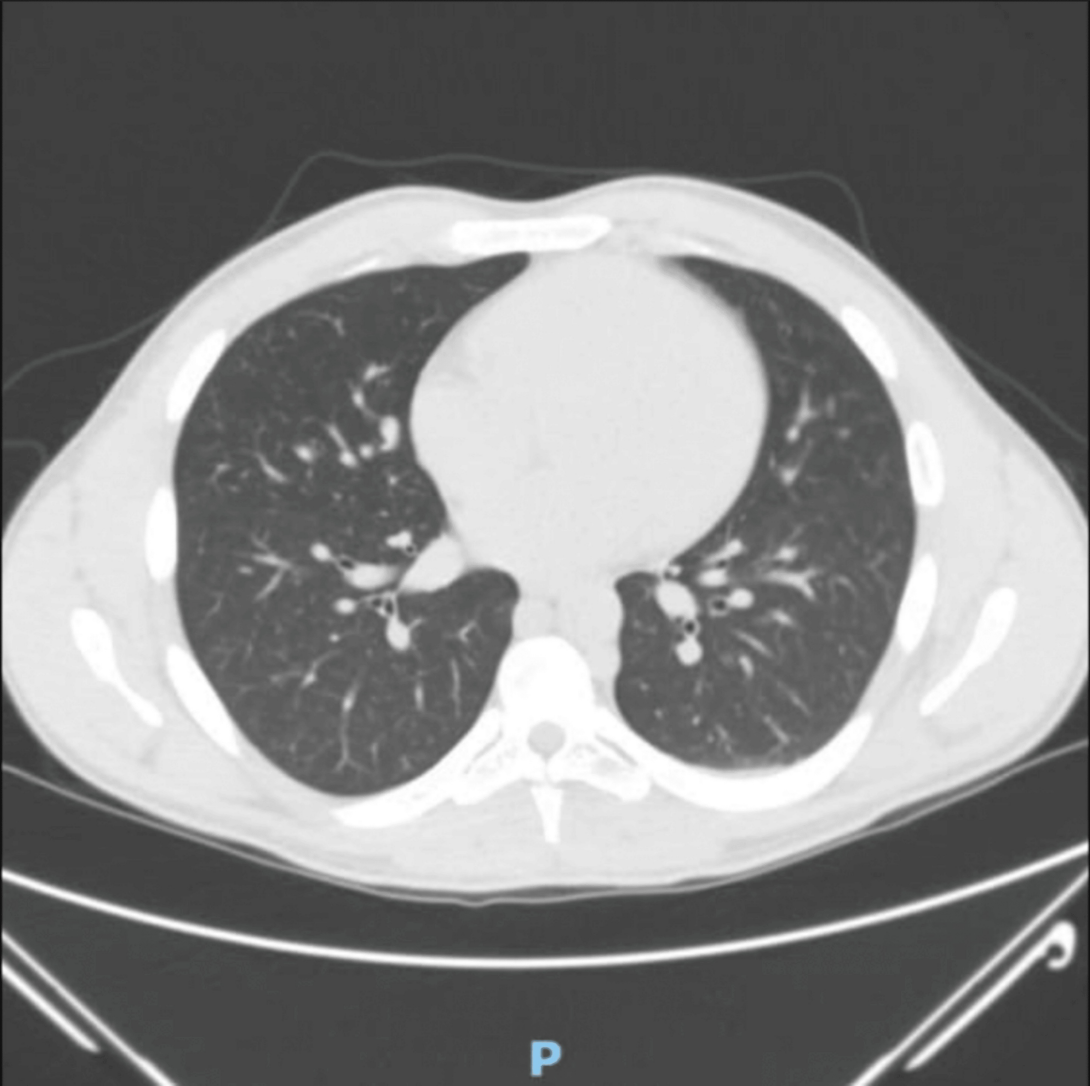
CT chest showing mild haziness

A previously healthy, 22-year-old male patient presented with a one-month history of high-grade intermittent fever and severe lower back pain, accompanied by unintentional weight loss. He had been hospitalized a week earlier for fever accompanied by a productive cough, during which a CT scan of the chest (Figure 1) revealed findings consistent with pneumonia. Despite empirical antibiotic therapy, his symptoms persisted, leading to readmission for further evaluation.

On examination, he was febrile and had a positive straight leg raise test, indicating low back pain, along with bilateral warmth of the upper thighs and neck stiffness. Chest and abdominal examinations were unremarkable. Given the warmth and bilateral calf muscle tenderness, D-dimer testing was performed and found to be markedly elevated.

Laboratory tests revealed significantly raised C-reactive protein (101 mg/L (reference: 0-5 mg/L) and D-dimer levels (>20), with normal white cell count. Hyponatremia was present (Na 129 mmol/L; reference: 136-145 mmol/L), along with mildly elevated creatine phosphokinase (CPK 413 U/L). Procalcitonin (0.07 ng/mL), HIV, and hepatitis panels were negative.

**Figure 2:**
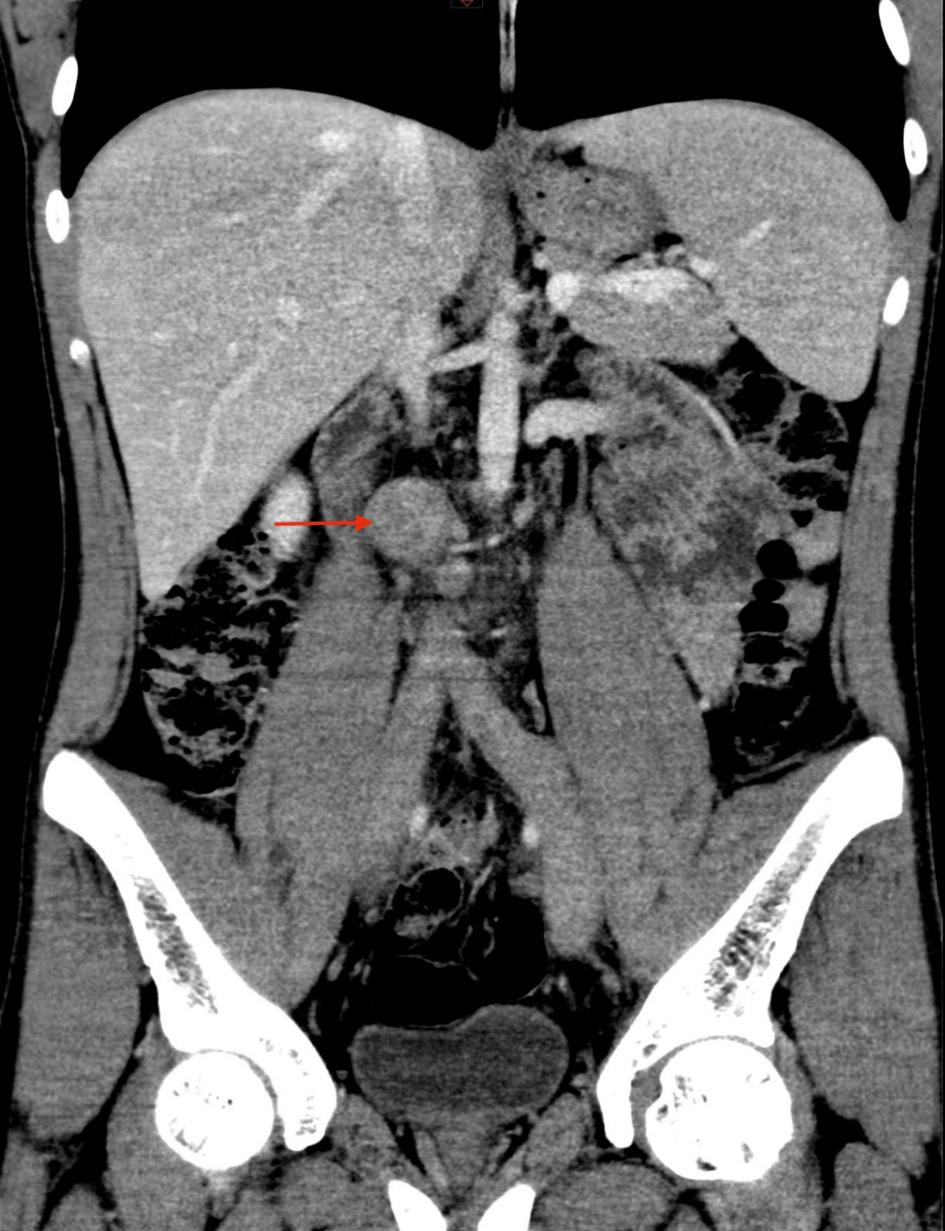
CT coronal image of the abdomen showing ectatic intrabdominal IVC with non-opacified lumen showing complete thrombus (red arrow) IVC: inferior vena cava

A CT abdomen without contrast showed faint opacification of the IVC, common, internal, and external iliac veins with an ectatic bulge in the infrahepatic IVC (Figure 2).

**Figure 3:**
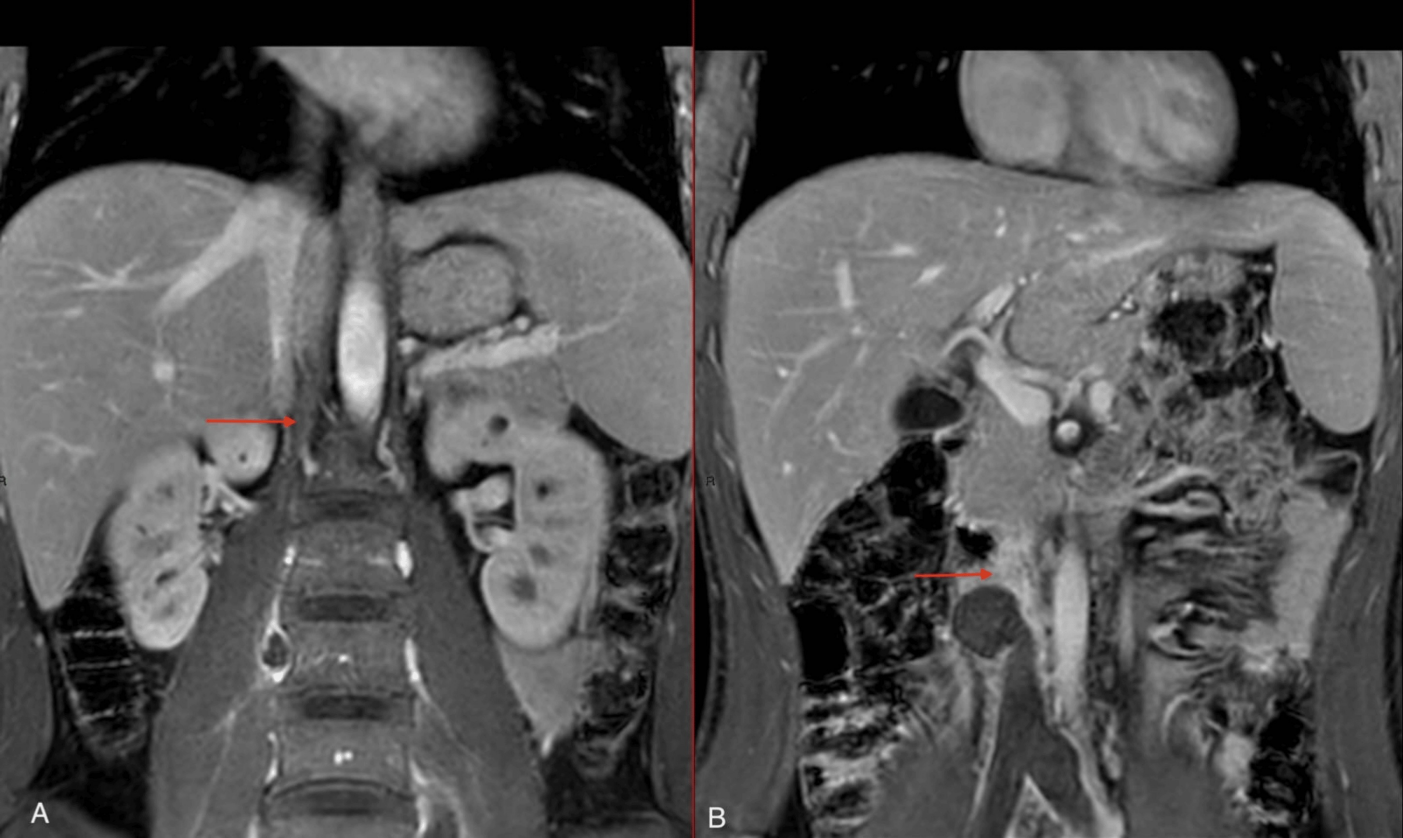
MRI abdomen with contrast - coronal view (A) Atretic infrahepatic and non-opacified IVC and bilateral right and left common iliac veins. (B) Image showing the small caliber of the infrahepatic IVC. IVC: inferior vena cava

**Figure 4:**
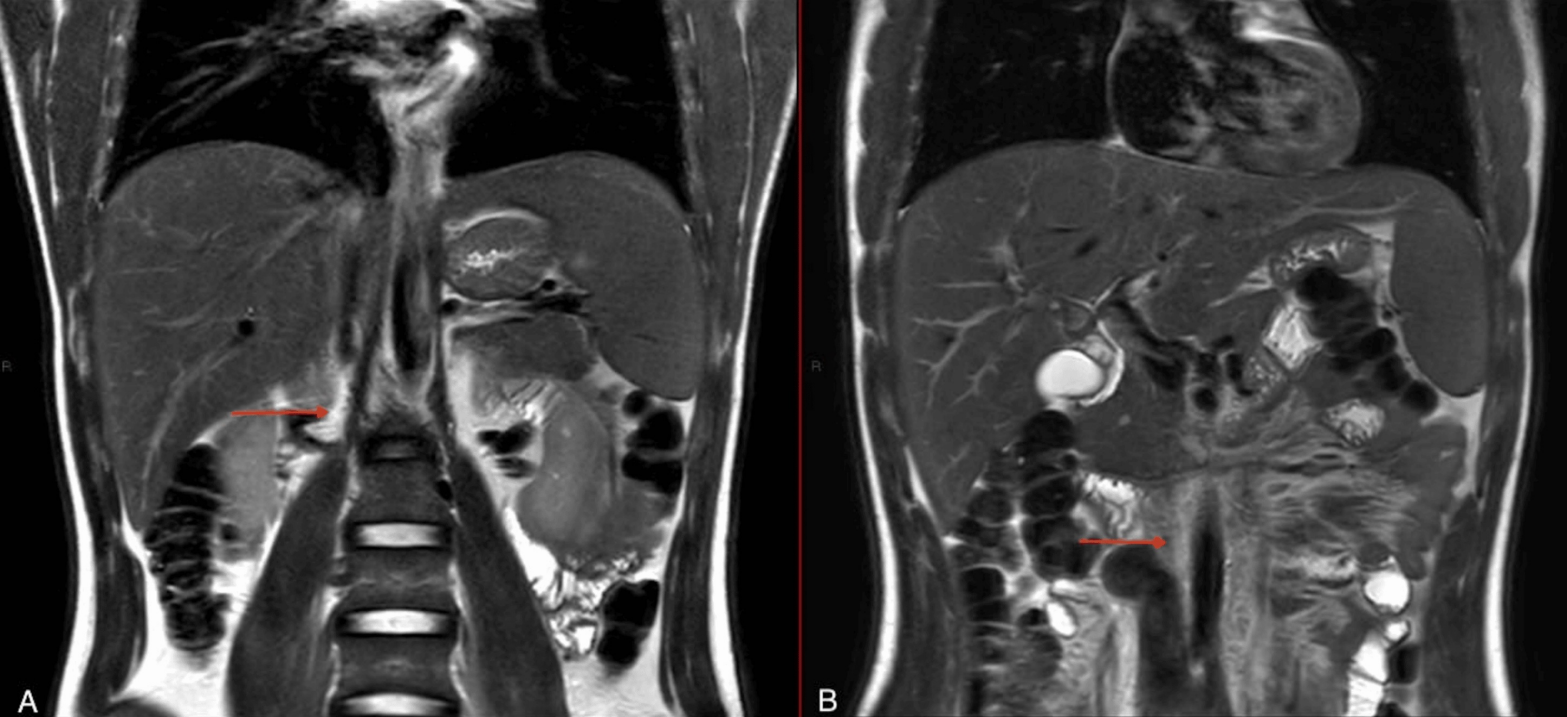
T2-weighted MRI abdomen without contrast - coronal view (A) Image showing atretic segment. (B) Image showing non-opacified dilated IVC. IVC: inferior vena cava

For further evaluation of the ectatic IVC segment and lack of contrast opacification, an MRI with and without contrast (Figures 3-4) of the abdomen was performed, which revealed atresia of the infrahepatic segment of the IVC with complete thrombosis of the intra-abdominal IVC, as well as the common, internal, and external iliac veins. Prominent collateral venous circulation was also noted.

**Figure 5:**
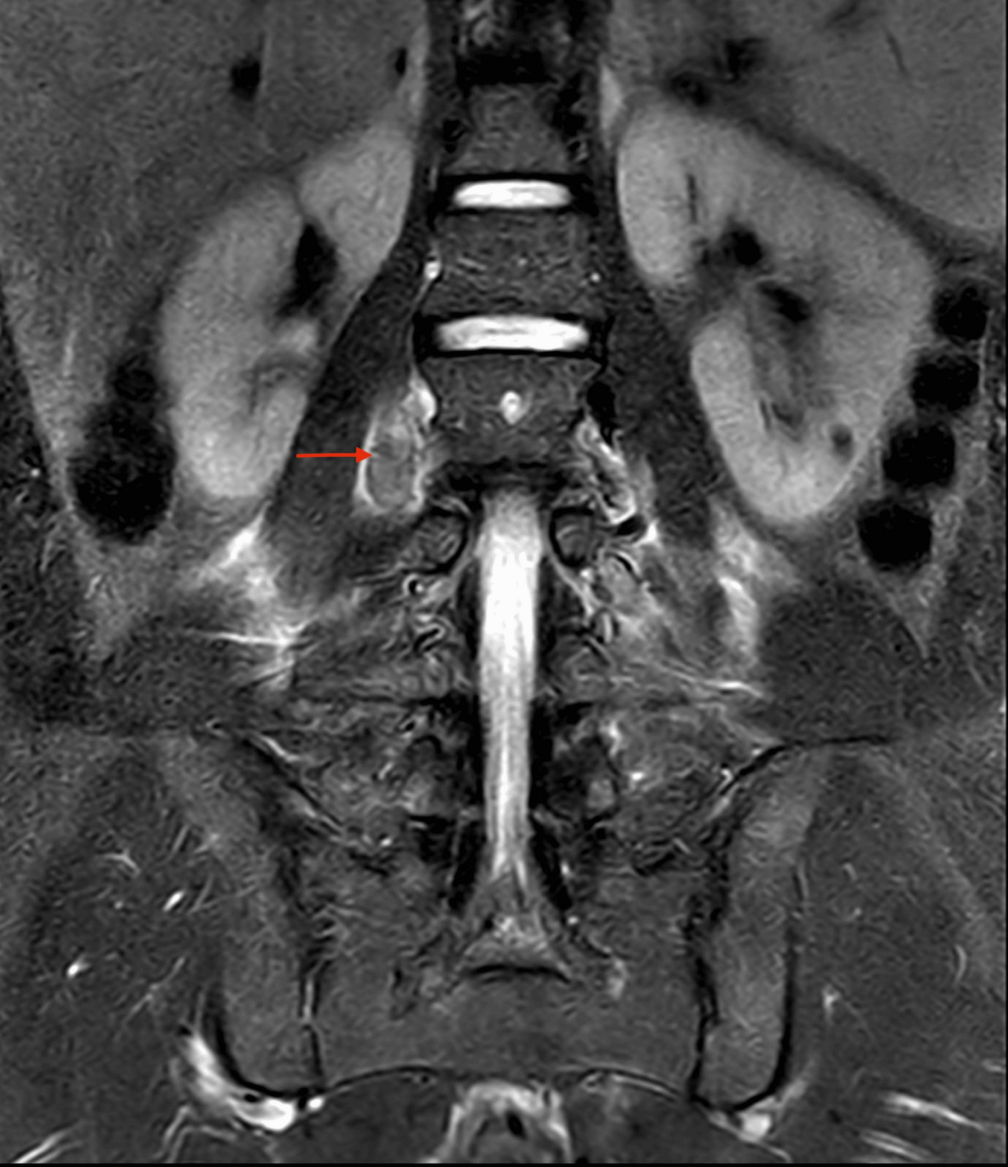
Lumbar spine MRI showed the presence of prominent collateral veins due to IVC atresia IVC: inferior vena cava

MRI of the lumbar spine (Figure 5) ruled out spinal pathology but supported the presence of prominent collateral veins due to IVC atresia. Echocardiography was normal. Genetic studies revealed an MTHFR gene mutation. 

**Figure 6:**
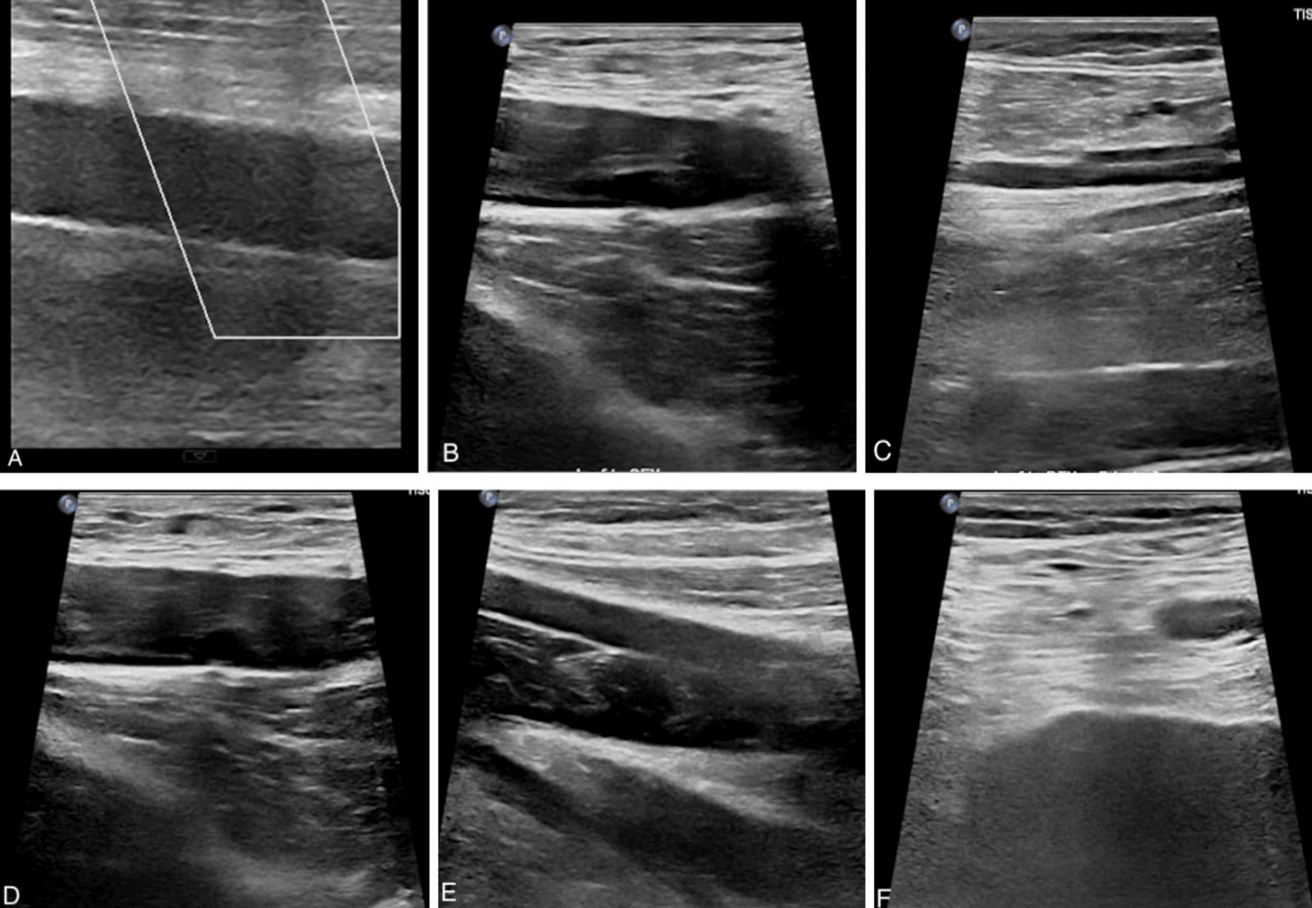
Venous ultrasound - greyscale images of the bilateral lower limbs veins showing echogenic thrombus (A) right common femoral vein; (B) right superficial femoral vein; (C) right popliteal vein; (D) left common femoral vein; (E) left superficial femoral vein; and (F) left popliteal vein.

Further to evaluate the extent of the thrombus, an ultrasound (Figure 6) of the bilateral lower limbs was done, which identified extensive DVT, which prompted further imaging. A vascular surgeon was consulted, and he advised a therapeutic dose of enoxaparin to be given. 

**Figure 7:**
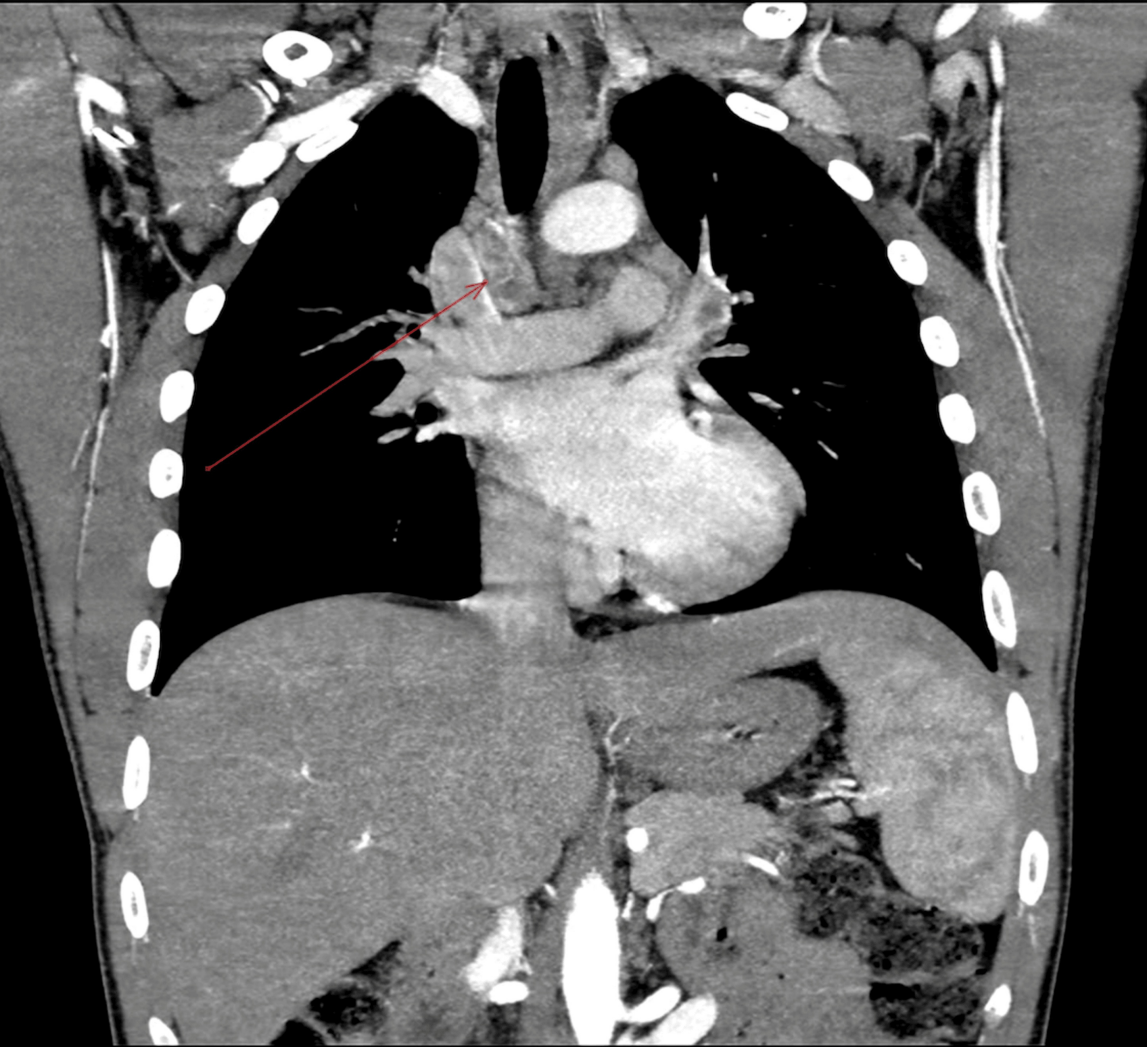
CT pulmonary angiography showing multiple necrotic mediastinal lymph nodes

He also advised a CT pulmonary angiogram (Figure 7) before discharge, which showed no evidence of pulmonary embolism; however, multiple necrotic mediastinal lymph nodes were identified, including a 3.4 x 2.0 cm subcarinal node, suggestive of either lymphoma or granulomatous disease.

**Figure 8:**
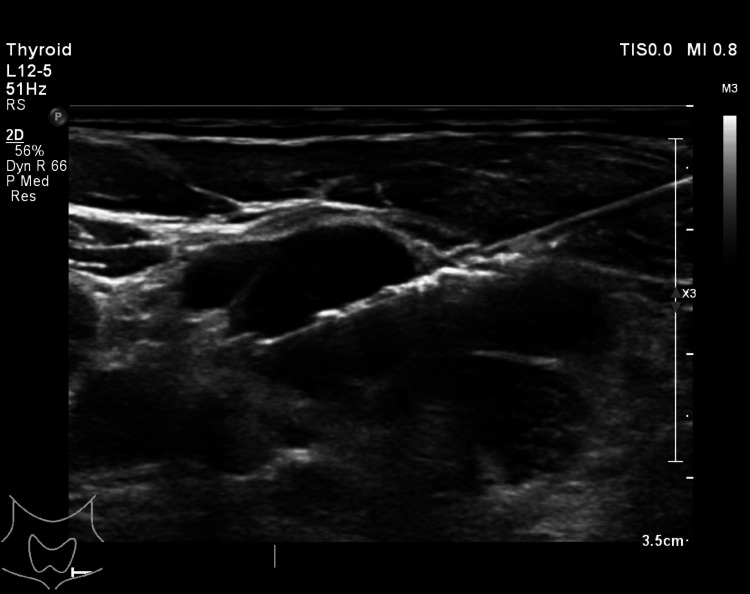
Ultrasound showing enlarged left cervical lymph node

Left cervical lymph node biopsy (Figure 8) was also done, and the histopathology report showed necrotizing granulomatous inflammation, which raised suspicion of tuberculosis or lymphoma because of the patient’s ongoing weight loss. A sputum test was performed to rule out tuberculosis, and a mediastinal lymph node biopsy was suggested to exclude lymphoma.

Further investigations were conducted to evaluate the cause of pyrexia of unknown origin. These included *Mycoplasma* IgG and IgM, Group A *Streptococcus* (GAS), influenza A and B, *Legionella*, COVID-19, rheumatoid factor, anti-CCP antibodies, malarial antigen, dengue IgG antibodies, *Brucella*, acid-fast bacilli (AFB) stain for tuberculosis, and blood cultures. p-ANCA and c-ANCA, JAK 2 mutation, Factor V Leiden, Antithrombin 3, Protein C and Protein S, Factor 2 (Prothrombin) were also done to rule out other causes of thrombosis. All the tests came back negative. Anticardiolipin antibodies were normal, which ruled out antiphospholipid syndrome and non-infective endocarditis.

The patient was managed with intravenous levofloxacin, anticoagulation therapy with a plan to transition to a direct oral anticoagulant at discharge, compression stockings, intravenous fluids, and close monitoring. The fever resolved after anticoagulants and antibiotics were started.

He was discharged on a therapeutic dose of rivaroxaban 15 mg twice a day for three weeks and was advised to return for a mediastinal biopsy, but he was lost to follow-up.

This case highlights the complexity of evaluating PUO in young adults, particularly in the presence of atypical features such as bilateral DVT and low back pain. It emphasizes the need to consider rare congenital vascular anomalies like IVC atresia as predisposing factors for extensive DVT and the importance of a thorough diagnostic approach, especially when radiological findings raise concern for underlying malignancy such as lymphoma.

## Discussion

This case presents a diagnostic challenge of a young male with PUO, extensive bilateral DVT, and eventual identification of IVC - a rare but significant vascular anomaly. The presence of constitutional symptoms, including fever, low back pain, and weight loss in a previously healthy individual initially raised a broad differential, encompassing infectious, malignant, autoimmune, and vascular causes.

The initial diagnosis of pneumonia, based on chest CT findings during the first admission, seemed plausible given the fever and productive cough. However, the patient’s lack of clinical improvement despite antibiotic therapy necessitated a broader diagnostic re-evaluation. PUO, traditionally defined as a fever >38.3°C lasting more than three weeks without a diagnosis after initial evaluation, remains a complex clinical scenario. The modern approach to PUO focuses on a tailored, imaging-driven diagnostic strategy to minimize unnecessary empirical treatments [1]. 

As part of this re-evaluation, the emergence of DVT in both lower limbs during readmission was a key clinical turning point. In young patients, unprovoked bilateral DVT is uncommon and should prompt investigation for underlying thrombophilia or anatomical abnormalities. Extensive thrombosis in a previously healthy individual without major provoking factors such as immobility, trauma, or malignancy led to the decision to obtain further vascular imaging.

Although rare, IVC atresia is a recognized congenital anomaly that may present with extensive lower extremity DVT in young adults without typical risk factors. Its incidence in the general population is 0.3-0.5% [4]. IVC atresia leads to impaired venous return and collateral vessel formation, predisposing individuals to thrombosis, especially under prothrombotic conditions. Our patient had markedly elevated D-dimer levels, and imaging confirmed extensive thrombosis with IVC atresia and collateral circulation, which explains the bilateral DVT and low back pain, often attributed to venous congestion or compression of adjacent structures [4,6], which provided a compelling anatomical explanation for the clinical presentation. Thus, what initially appeared to be an incidental vascular finding quickly became central in the diagnostic hierarchy, offering a unifying etiology for multiple symptoms.

Multiple contributing factors can underlie IVC thrombosis. Congenital IVC anomalies such as atresia or hypoplasia are important structural causes, especially in young individuals presenting with extensive or bilateral DVT in the absence of typical risk factors [7]. Additionally, inherited thrombophilias, including factor V Leiden, prothrombin G20210A mutation, and protein C or S deficiency, can significantly increase the risk of venous thromboembolism by altering normal hemostatic balance [8]. 

In this case, the presence of an MTHFR gene mutation adds further complexity. While the clinical significance of MTHFR polymorphisms (particularly C677T) remains controversial, they may contribute to hyperhomocysteinemia, especially in the presence of folate deficiency, thereby modestly increasing thrombotic risk [9]. A 2020 meta-analysis suggested that the association between MTHFR variants and venous thrombosis is likely influenced by both genetic background and environmental factors [10]. 

The incidental finding of mediastinal lymphadenopathy, particularly necrotic and bulky subcarinal nodes, expanded the differential diagnosis to include lymphoma and granulomatous diseases such as tuberculosis or sarcoidosis [11]. This finding raised concern for concomitant pathology, influencing the decision to pursue tissue diagnosis via biopsy, especially given the weight loss and systemic symptoms 

This case illustrates the interplay of three overlapping factors-congenital IVC atresia, possible genetic thrombophilia, and systemic inflammation - that acted synergistically to produce extensive thrombosis. Acquired prothrombotic conditions such as infection, inflammation, malignancy, and prolonged immobility can also serve as triggers, particularly when superimposed on congenital or genetic vulnerabilities [12]. 

Hyponatremia was likely multifactorial, with probable contributions from syndrome of inappropriate antidiuretic hormone secretion (SIADH) secondary to pneumonia and systemic inflammation, and possibly central nervous system involvement. It developed during hospitalization and improved with infection control.

The case highlights the value of advanced imaging in delineating vascular anatomy and identifying potential underlying causes, including malignancy. Earlier targeted imaging could have expedited diagnosis, reduced empirical treatment duration, and guided focused management sooner. The presence of low back pain with a positive straight leg raise test may be misleading, commonly suggesting lumbosacral radiculopathy; however, in this context, it was secondary to thrombosis of the veins of the lower limbs extending to the IVC [13]. Management of such patients requires a multidisciplinary approach involving vascular surgery, oncology, and infectious disease specialists. Anticoagulation remains the mainstay of therapy for extensive DVT, and long-term management decisions must consider the chronic nature of the vascular anomaly.

## Conclusions

In summary, a high index of suspicion of underlying pathology including congenital IVC anomalies and genetic disorders should be considered in young patients presenting with bilateral DVT. The coexistence of congenital IVC atresia and MTHFR mutation is a rare but significant risk factor for extensive DVT, especially in young adults. This case also demonstrates how the diagnostic focus can shift from more common infectious or malignant causes to rare structural anomalies when the clinical picture evolves. Additionally, this case also illustrates the diagnostic challenge of concurrently evaluating systemic causes such as granulomatous or lymphoproliferative disorders. The clinical course emphasizes the need for a balanced, systematic, and multidisciplinary approach in PUO cases with thrombosis, integrating work-up for congenital vascular anomalies alongside investigation for infectious, inflammatory, and malignant etiologies, especially when constitutional symptoms persist and imaging raises concern for underlying systemic disease.
